# Immune cell impact of three differently coated lipid nanocapsules: pluronic, chitosan and polyethylene glycol

**DOI:** 10.1038/srep18423

**Published:** 2016-01-05

**Authors:** Cristiano Farace, Paola Sánchez-Moreno, Marco Orecchioni, Roberto Manetti, Francesco Sgarrella, Yolande Asara, José M. Peula-García, Juan A. Marchal, Roberto Madeddu, Lucia G. Delogu

**Affiliations:** 1 Department of Biomedical Science, University of Sassari, 07100 Sassari, Italy; 2 Biocolloid and Fluid Physics Group, Department of Applied Physics, University of Granada, 18071 Granada, Spain; 3 Department of Chemistry and Pharmacy University of Sassari, 07100 Sassari, Italy; 4 Department of Clinical Medicine and Experimental Oncology, University of Sassari, 07100 Sassari, Italy; 5 Department of Applied Physics II, University of Málaga, 29071 Málaga, Spain; 6 Biopathology and Medicine Regenerative Institute (IBIMER), University of Granada, 18100 Granada, Spain; 7 Biosanitary Institute of Granada (ibs.GRANADA), University Hospitals of Granada-Univesity of Granada, 18071 Granada, Spain; 8 Department of Human Anatomy and Embryology, University of Granada, 18012 Granada, Spain; 9 National Institute of Biostructures and Biosystem (INBB), Rome, Italy

## Abstract

Lipid nanocapsules (NCs) represent promising tools in clinical practice for diagnosis and therapy applications. However, the NC appropriate functionalization is essential to guarantee high biocompatibility and molecule loading ability. In any medical application, the immune system-impact of differently functionalized NCs still remains to be fully understood. A comprehensive study on the action exerted on human peripheral blood mononuclear cells (PBMCs) and major immune subpopulations by three different NC coatings: pluronic, chitosan and polyethylene glycol-polylactic acid (PEG) is reported. After a deep particle characterization, the uptake was assessed by flow-cytometry and confocal microscopy, focusing then on apoptosis, necrosis and proliferation impact in T cells and monocytes. Cell functionality by cell diameter variations, different activation marker analysis and cytokine assays were performed. We demonstrated that the NCs impact on the immune cell response is strongly correlated to their coating. Pluronic-NCs were able to induce immunomodulation of innate immunity inducing monocyte activations. Immunomodulation was observed in monocytes and T lymphocytes treated with Chitosan-NCs. Conversely, PEG-NCs were completely inert. These findings are of particular value towards a pre-selection of specific NC coatings depending on biomedical purposes for pre-clinical investigations; *i.e.* the immune-specific action of particular NC coating can be excellent for immunotherapy applications.

Nanomedicine has reached the interest not only of the scientific community but also of the public, becoming one of the most promising approaches for developing new tools in clinical practice^1^,^2^. Among other nanomaterials, biodegradable lipid nanocapsules (NCs) present incredible characteristics as drug carriers or in diagnosis applications as contrast agents^3^,^4^. Their useful properties include biocompatibility and biodegradability^5^, the ability to perform a controlled release of drugs^6^,^7^ and to target specific tissues^8^. Specifically, NCs consisting of an oil-filled core with a surrounding polymer shell can be used to encapsulate and deliver hydrophobic drugs^9^,^10^. The appropriate carrier design and functionalization, particularly the composition and surface properties, are essential to ensure high biocompatibility and to protect molecules of interest from degradation and premature elimination^11^. Biodegradable polymers and molecules have been extensively studied as loading molecules for NCs to improve their hydrophilicity in biological media, for new possible treatments of many diseases. Well before any pre-clinical application, it is of fundamental importance to choose the most suitable coating for the NCs. Moreover, for any medical application which requires intravenous injection, the first type of cells that will interact with the NCs are the blood immune cells making the NCs immunocompatibility assessment of critical importance for any translation into clinical practice. Aiming at providing an extensive overview on the immune impact of differently functionalized NCs, we report for the first time a comprehensive analysis on immune cell interaction with three different NCs coatings: pluronic F68 (Pluro), chitosan (Chito) and polyethylene glycol-polylactic acid (PEG-PLA, indicated in the text as PEG). Pluro, Chito and PEG coatings have been successfully used for NC functionalization for many applications^12^,^13^. PEG has been significantly employed to functionalize several nanomaterials to better deliver different genes and drugs such as camptothecin for the cancer treatment^14^,^15^,^16^,^17^. Controversial studies have been published in literature on the ability of PEG coating to be internalized into cells. Some studies have already reported the capability of PEG coating to be internalized into macrophages and other cells such as hepatocytes^18^,^19^. However, very recently Yang Q and colleagues have shown a reduced uptake of PEG coated nanoparticles by macrophages, but these interactions with phagocytic cells are critically dependent on the conformation of individual PEG chains and on the brush conformation onto the particles. Furthermore, very few results were reported about internalization of PEG coating into other immune cells subpopulations^20^. NCs loaded with chitosan have been extensively studied to enhance the therapeutic use of siRNAs^12^,^21^. Moreover, chitosan is commonly used as a transacylation polymer evidencing its non-toxicity^12^,^21^,^22^. In order to improve the NC drug delivery abilities, the NC shell can be also functionalized with pluronic, a nonionic triblock copolymer. Thus its amphiphilic structure can be used to increase the water solubility of many substances. For this reason, pluronic coated NCs have been evaluated for various drug delivery applications, *i.e.* in cancer cells^13^. Moreover, these nanocarriers have been shown to inhibit multiple drug resistant proteins (MDR) and other drug efflux transporters on the surface of cancer cells; MDR proteins are responsible for drug efflux from cells and hence decrease the susceptibility of cancer cells to chemotherapeutic agents such as doxorubicin^23^. All these substances have been successfully used to improve the ability of NCs to deliver drugs and genes for the treatment of cancer and other diseases.

Biocompatibility analyses of NCs have been previously conducted, focusing on different organs and evidencing low toxicity^24^. However, to the best of our knowledge no studies reported a comprehensive immune-compatibility comparison analysis of differently functionalized NCs on immune cells, specifically *ex vivo* from healthy donors. For any translational application of nanomaterials in medicine, a key step is represented by the assessment of their possible interactions with the immune system, independently of their specific purpose^25^,^26^,^27^,^28^. In this context, the lack of information in literature created an urgent need to better understand different NC-coating effects on the immune system.

Hence, here the impact of three differently functionalized NCs was analyzed, namely Pluronic, Chitosan, and PEG, on human primary immune cells populations, in particular on red blood cells (RBCs) hemolysis and peripheral blood mononuclear cells (PBMCs) from healthy donors. First, we deeply analyzed the possible differences in NC internalization on PBMCs and subpopulations, such as T, B, NK cells and monocytes, to assess whether a certain coating could be more suitable for the cell uptake. We explored the possibility of necrosis, apoptosis and proliferation of PBMCs and subpopulations of T cells and monocytes, as representative of the adaptive and innate immune response. The functionality effect was tested observing: (i) the cell diameter modification; (ii) the expression of the most critical activation marker, namely CD25 and CD69; (iii) the production of a wide variety of cytokines.

## Results

### Nanocapsule synthesis and characterization

**Figure 1:**
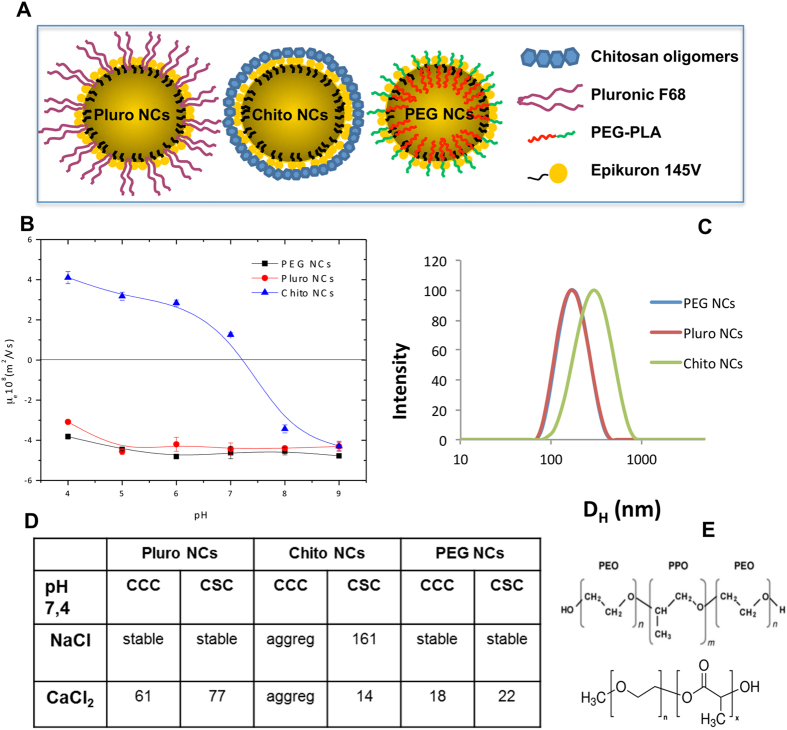
Nanocapsule characterization: (A) Schematic details of the nanocapsule composition. (**B**) Electrophoretic mobility vs. pH of nanocapsules. (**C**) Intensity-weighted size distribution of NCs obtained from a DLS measurement. (**D**) Critical coagulation concentration (CCC) and critical stabilization concentration (CSC) data (mM), at pH 7.4, using NaCl and CaCl_2_ as aggregating salts. (**E**) Chemical structure of Pluronic F68 (average MW 8400 g/mol) with a central hydrophobic fragment of polyoxypropylene (PPO) and identical hydrophilic chains of polyoxyethylene (PEO) at both sides. n = 75 PEO units and m = 30 PPO units (upper image). Chemical structure of PEG-PLA. PEG average MW 350 g/mol (n=8EO units). PLA average MW 1000 g/mol (lower image).

Two nanocapsule systems, indicated as EPI and CS in Sánchez-Moreno *et al.*^8^, were previously obtained and characterized with an anionic (Pluro NCs) and a cationic surface (Chito NCs); instead, a new nanocapsule system (PEG NCs) was prepared by a solvent-displacement technique following the procedure of Prego *et al.*^29^ by mixing an organic and an aqueous phase. Figure 1A shows a scheme of the nanocapsule composition. All nanosystems were formed by olive oil in their hydrophobic core and epikuron on their shell; depending on the composition of the aqueous phases (pluronic F68, chitosan or PEG-PLA), the final samples showed different shell composition and thus, different interface properties. The nanocapsule size and electrophoretic mobility were characterized by dynamic light scattering (DLS) while the colloidal stability was spectrophotometrically studied working at physiological pH in simple saline media (NaCl and CaCl2) by analyzing the change of particles diameter as a function of time.

The hydrodynamic size of all nanocapsule systems showed a narrow distribution (PDI < 0.15), as it is illustrated by the representative intensity weighted size distribution of different NCs reported in Fig. 1C. In the present study, the synthesis procedure yielded to spherical nanocapsules with an average diameter in the nanometric scale that is potentially useful for biological applications; in fact, a mean diameter under 200–300 nm is advised in specialised literature. The size of PEG NCs was 167 ± 14 nm, while the size of Pluro NCs and Chito NCs were 170 ± 20 nm and 340 ± 30 nm, respectively, as previously reported^8^. The electrophoretic mobility data of PEG NCs, gathered from low-ionic-strength media, are also shown in Fig. 1E. The μ_e_ results agree with the nature of the shell and they confirm the presence of the different molecules used in their synthesis. Pluro and PEG NCs showed a typical behaviour of colloids with weak acid groups, which come exclusively from the epikuron molecules with phosphatidyl-choline as the major component, giving lower μ_e_ values at acidic pH values than those found at neutral and basic pH^30^,^31^. PLA chains absorb onto hydrophobic interfaces, while PEG hydrophilic chains remain extended in the hydrophilic phase. Since PEG is a non-ionic surfactant it does not alter the electrical state of the surface. A similar pattern was observed for Pluro NCs, in which the non-ionic polyethylene oxide chains from pluronic did not influence the electric surface behaviour. On the other hand, the μ_e_ behavior of Chito NCs radically changed as a consequence of the presence of chitosan chains with glucosamine groups, presenting with a weak basic character and driving mobility from positive values at acidic pH to negative values at basic pH^32^.

The analysis of coagulation kinetics enabled us to calculate the stability factor W, which provides information on the coagulation probability (W = 1 indicates a completely unstable system, while W = ∞ means total stability); at the same time, nanoparticle critical coagulation concentration (CCC) and critical stabilization concentration (CSC) were obtained, which are considered fundamental parameters in colloidal-stability studies. A thorough explanation of the respective significances of these values and on the method applied to obtain CCC and CSC values from coagulation kinetics data, is given in the supplementary section of reference^33^. The CCC value is related to destabilization processes and it indirectly gives information on the surface-charge density of the particles; thus, a low CCC means low stability caused by a low surface-charge density. Instead, the CSC value – defined as the minimum salt concentration at which the system begins to re-stabilize when salinity is increased– is associated with the surface hydrophilicity. This kind of restabilization phenomenon at high salt concentrations is well known in hydrophilic colloidal systems and it is governed by hydration forces^34^. Figure 1D shows the CCC and CSC values of different NC systems. With respect to the stability of the PEG system in NaCl, no aggregation of the particles was detected even at concentrations up to 4M because the CCC and CSC concentrations overlapped, inducing completely stable systems. An analysis of the colloidal stability in CaCl_2_ indicated that Chito NCs were the least stable of the systems^8^ since they coagulated as soon as the nanocapsules were immersed in the buffer at pH 7.4. In a high-salinity solution this system was stable due to the action of the repulsive hydration forces due to the fact that chitosan in an hydrophilic material. PEG NCs presented lower CCC value (22 mM) compared to Pluro NCs (61 mM), since the number of polyethylene oxide (PEO) fragments in the pluronic (75 units) was higher compared to the number of ethylene oxide (EO) in the PEG (8 units) as shown in Fig. 1B; in conclusion, these molecules contribute to the stabilization of the systems by means of a steric mechanism and increase the surface hydrophilicity^32^,^35^. The CSC data corresponding to CaCl_2_ confirm the presence of pluronic (in Pluro NCs) and PEG (in PEG NCs) molecules on the surface, showing a restabilization process typical of surfaces with hydrophilic character. Nanoemulsion behaviour in several fluids (water, PBS and DMEM with 10%FBS) was also evaluated, revealing high stability in all tested solutions (data not shown).

### Functionalized nanocapsule biocompatibility in red blood cells and immune cells

**Figure 2:**
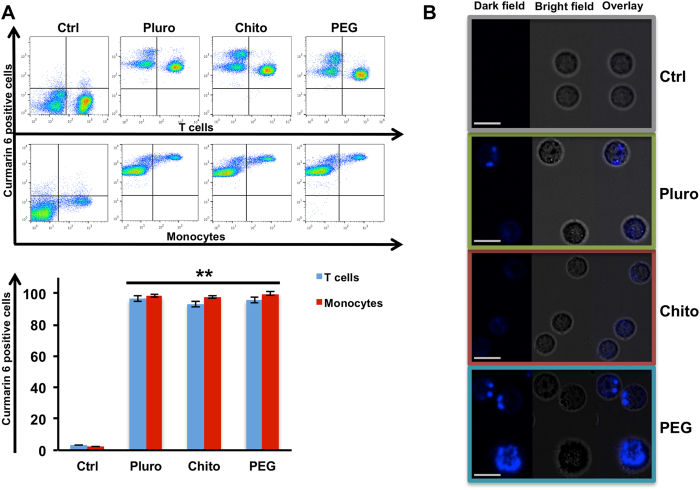
Uptake analysis of Nanocapsules. (**A**) T cells (CD3+) and Monocytes (CD14+) uptake of Pluro, Chito, PEG nanocapsules loaded with Coumarin 6. Samples were analysed by FACS Calibur (BD Biosciences) and FlowJo software. On the upper pseducolor Dot Plot are representative experiments out of 4 independent investigations. Down **=P value < 0,01. (**B**) Confocal microscopy imaging of PBMCs incubated with Pluro Chito and PEG NCs loaded with coumarin 6 at the concentrations of 1 × 10 e11 mL^−1^. Dark field, bright field and overlay images were captured with Leica TCS SP5 confocal (Leica). Scale bar 25 μm.

The release of hemoglobin from damaged RBCs was observed and any NC-related damage on red blood cells was excluded (S. Fig. 1). To assess the immune cell internalization ability of NCs, they were encapsulated with coumarin 6 as a fluorophor in the hydrophobic cores of Pluro, Chito and PEG NCs. A dose-response analysis of the nanoparticle uptake at 1 × 10^10^NCs mL^−1^, 1 × 10^11^NCs mL^−1^ and 1 × 10^12^ NCs mL^−1^ was performed (S. Fig. 2A). The ability of differently functionalized NCs to enter inside the cells was described through an accurate analysis by flow cytometry^33^ and confocal microscopy. Moreover, it has been previously determined that after few hours of exposure, the amount of adhering particles is in most cases minimal in comparison to the active uptake and the internalized load^36^. The performed time response showed no uptake difference in the three analyzed time points (at 6, 12 and 24 hours) (S. Fig. 2B). Furthermore, the Trypan Blue washing was performed to quench the NC signal outside the cells, as we previously performed for other nanomaterials^17^ (S. Fig. 2C). The 24-hour incubation time was adopted for all the other investigation since this is a good time point to study the effect of any nanoparticle or nanomaterial on immune cells, as other research groups suggested^37^,^38^.

Data shown at 1 × 10^11^NCs mL^−1^ and at 1 × 10^12^ NCs mL^−1^ after 24-hour incubation that almost 100% of cells internalized all three NCs (S. Fig. 2A).

Intriguingly, during the present study no inhibition was observed in NC internalization mediated by PEG (Fig. 2A and S. Fig. 2A,B). The PEG mediated action on internalization is still under discussion due to its well-known stealth property^20^. However, other recent studies evidenced how a specific PEG coating can enhance the nanoparticle uptake, such as in the case of cancer cells^39^,^40^. On the other hand, the chosen time point (24 h) might be also responsible for the no PEG mediated uptake inhibition, as it was also reported in the study of Sheng Y *et al.*^41^. The concentration chosen for the further experiments was 1 × 10^11^NCs mL^−1^; indeed, a high uptake for all NC coatings was found at this amount of particles, ensuring that any interaction into immune cells, upon internalization, was missed. Moreover this concentration was previously found to be optimal for drug delivery studies^42^.

To confirm the preliminary uptake data, the cell internalization in the subpopulations of immune cells was detected.

**Figure 3:**
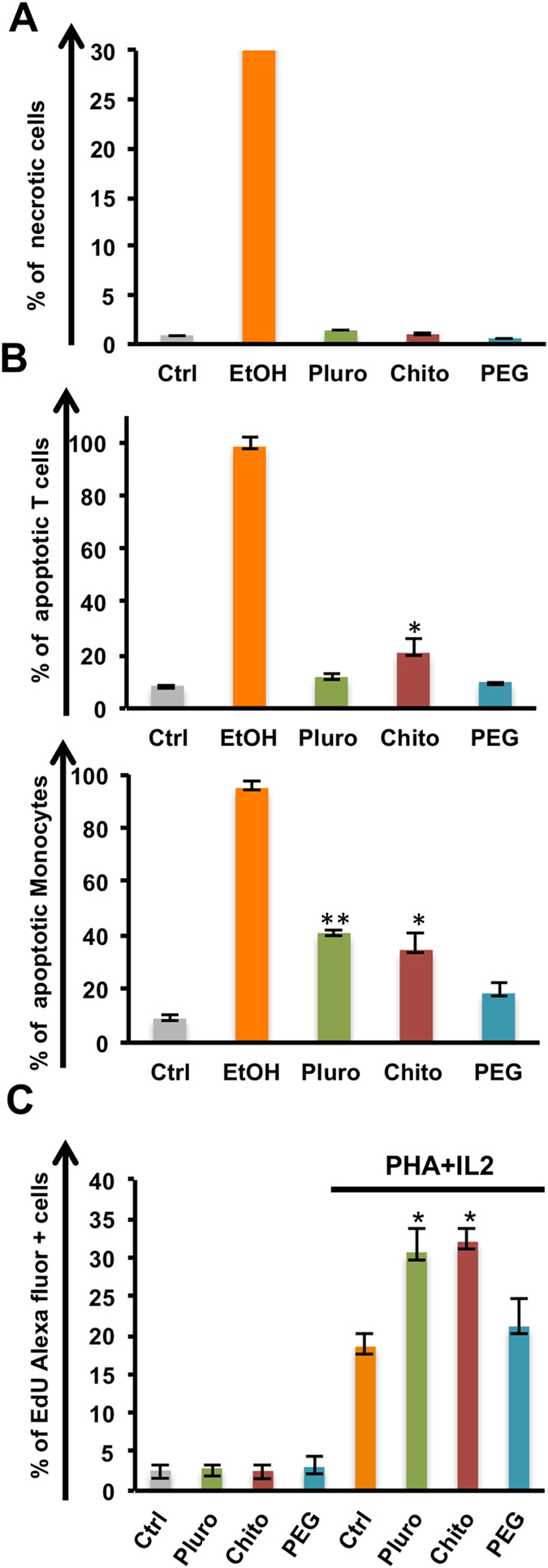
Viability and proliferation. PBMCs were incubated with Pluro, Chito and PEG NCs at the concentration of 1 × 10 e 11 mL^−1^ or left untreated (Ctrl). (**A**) Percentage of necrotic cells was assessed by a Live/Dead staining after 24 hours of incubation, ethanol (70%) was used as positive control. (**B**) Apoptosis was assessed using Annexin V staining for Monocytes (CD14+ cells) and T cells (CD3+ cells), ethanol (70%) was used a positive control. Samples were analyzed by FACS Calibur (BD Biosciences). (**C**) Proliferation assay was performed on stimulated cells with phytohemagglutinin 2% (PHA) and interleukin 2 (IL2) 30 u/mL for three days. 5-ethynyl-2´-deoxyuridine (Edu) is incorporated into DNA during active DNA synthesis; Percentage of Edu positive cells is reported. All experiments were performed at least in triplicate in independent assays (*=P value < 0.05, **=P value < 0.01).

Cells were gated by specific membrane markers for the two major subpopulations of PBMCs, T cells and monocytes (Fig. 2A). All types of NCs were able to target T cells and monocytes with a high frequency close to 100%, as showed by the FACS pseudocolor Dot Plot and by histograms. The uptake analysis was tested also on a wide variety of other subpopulations, such as lymphocytes T helper, lymphocytes T cytotoxic, natural killer cells and B cells gated by different surface marker staining by flow cytometry (S. Fig. 3); again, NCs were able to enter into all studied cell types with a passive targeting of immune cells.

Moreover, the internalization of Pluro, Chito and PEG NCs was confirmed by confocal microscopy. PBMCs were treated with NCs at the concentration of 1 × 10^11^NCs mL^−1^ for 24 hours and then visualized. All pictures confirmed the internalization of Pluro, Chito, PEG NCs into cells (Fig. 2B). This data gives further proof of the great potential of NCs to perform intracellular drug delivery^3^,^6^,^33^,^43^,^44^,^45^. The impact of the differently functionalized NCs on cell viability was studied performing a live/dead staining. PBMCs were either incubated for 24 hours with the NCs or left untreated; positive control was set up by ethanol incubation.

Upon exposure to the three types of NCs, no significant necrotic cell levels were noticed (Fig. 3A). The early apoptotic cells with Annexin V staining on T lymphocytes and monocytes were then observed. Flow cytometry analysis showed a different impact on cells NC-coating dependent (Fig. 3B). The Annexin-V staining evidenced that the NC functionalization could influence the viability on different cell types. Similar results for both Pluro and PEG NC functionalizations were found in lymphocytes: no statistically significant differences were observed between the control and the T lymphocytes treated with NCs. On the other hand, the incubation with Chito-NCs induced cell apoptosis in T lymphocytes (P value < 0.05). A similar action of Chito-NCs was also assessed against monocytes. The apoptosis induction in monocytes was more than three times higher compared to controls. Moreover, in this type of cells also pluro-NCs were responsible for a clear induction of apoptosis (P value < 0.01). Cell viability was not affected by PEG-NCs in both cell populations (Fig. 3B). These different actions exerted by Pluro and Chito-NCs were also confirmed by the proliferation assay on total PBMCs (Fig. 3C).

Samples were treated with phytohemagglutinin at 2% and 30 μ/mL of IL2 for three days to boost the proliferation in presence of lipid nanocapsules. Control samples were left untreated. A staining with 5-ethynyl-2´-deoxyuridine (Edu) alexafluor conjugated was performed; Edu was incorporated into DNA during active DNA synthesis. Interestingly, Pluro and Chito NCs induced an enhancement of proliferation compared to controls (P < 0.05). PEG-NCs confirmed the viability data; in fact, PEG-NCs treated samples were perfectly comparable with controls, showing the total absence of cell induction.

### Immune cells activation analysis

**Figure 4:**
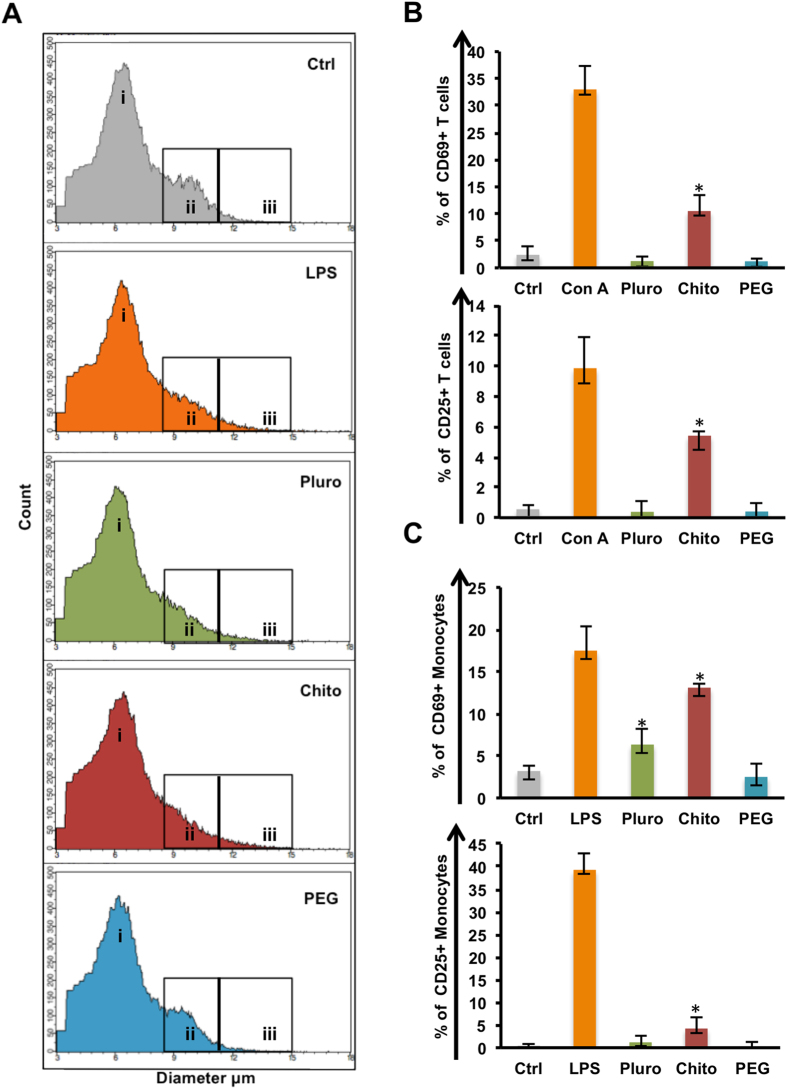
Activation assays. PBMCs were incubated with Pluro, Chito, PEG NCs at the concentration of 1 × 10 e 11 mL^−1^ or left untreated (Ctrl) for 24 h. Lipopolysaccharides (LPS 2μg/mL) or concanavalin A (ConA, 10 μg/mL) were used as positive controls. (**A**) Morphological analysis (count and diameter) of PBMCs with Scepter 2.0 (Millipore) i highlights the lymphocytes peaks boxes, ii highlights monocyte peaks and iii highlights activated monocytes with a diameter higher than 11.25 μm. Percentage of CD25 and CD69 cell surface activation marker expression on T cells (**B**) and monocytes (**C**) were analyzed by flow cytometry. Experiments were performed at least in triplicate (*p value < 0.05).

The different action of Pluro, Chito and PEG NCs on the immune cell functionality was analyzed by two activation assays (Fig. 4). Firstly, cell morphological changes in terms of diameter after NC incubation were observed. In fact, when immune cells are under activation stimuli a change in diameter occurs by increasing the cellular volume. For instance, activated monocytes increase their diameter starting phagocytosis. In addition, the naïve T cells before the contact with the antigen have a resting diameter of 6–8 μm, in our case the same role can be played by NCs; on the other hand, activated T cells increase their diameter of 12 μm or more, thanks to the induction of the replication cycle^46^. This is the criterion used to deeply analyze the immune impact of NCs and to further validate data found with Flow Cytometry. The present study was carried out on PBMCs from healthy donors that included different cell populations, T cells, B cells, monocytes, dendritic cells and natural killer cells to better reflect the effects on the pool of cell populations relevant to *in vivo* immune responses.

PBMCs from healthy donors were treated with the three NCs at the intermediate concentration of 1 × 10^11^ mL^−1^; experiments and data analysis were carried out with Scepter (Millipore). Figure 4A shows the cell diameter of monocytes (Fig. 4A ii) after treatment with Pluro and Chito NCs, compared to controls; in particular, Chito NCs led to similar changes in monocyte diameter compared to lipopolysaccharide (LPS) treated samples. Thus, cells with a diameter of 2.52 × 10^4^ larger than 11.75 μm were found in Chito NCs compared to cells of 2.33 × 10^4^ in LPS; the relative amount of cells in control samples was 1.35 × 10^4^ (Fig. 4A iii). Intriguingly, PEG-NCs samples were perfectly comparable with controls having 1.08 × 10^4^ cells, without any activation stimuli (Fig. 4A iii).

Afterwards, the different immune activation mediated by Pluro, Chito, PEG NCs was analyzed by observing the modulations of CD25 and CD69 cell surface activation markers on T cells and monocytes. CD25 and CD69 are critical markers for the immune response. CD25 (alpha chain of the IL-2 receptor) is a late activation antigen. Whereas, the CD69, a member of the C-type lectin superfamily (Leu-23), is one of the earliest cell surface antigens expressed by immune cells following activation. PBMCs were treated with the three NCs at the intermediate concentration of 1 × 10^11^ mL^−1^. Interestingly, CD25 and CD69 expression on T cells and monocytes was not modulated by PEG NCs (Fig. 4B,C), evidencing no induced activations on both analyzed cell types. Likewise, Pluro NCs did not boost the expression of activation markers on T cells (Fig. 4B). However, Pluro NCs were found able to induce CD69 expression on monocytes (Fig. 4C), highlighting the Pluro NCs specific action on this cell population. Notably, Chito NCs were able to strongly activate the immune cells, giving a powerful CD25 and CD69 modulation on both immune cell types (Fig. 4B,C).

### Cytokine secretion

**Figure 5:**
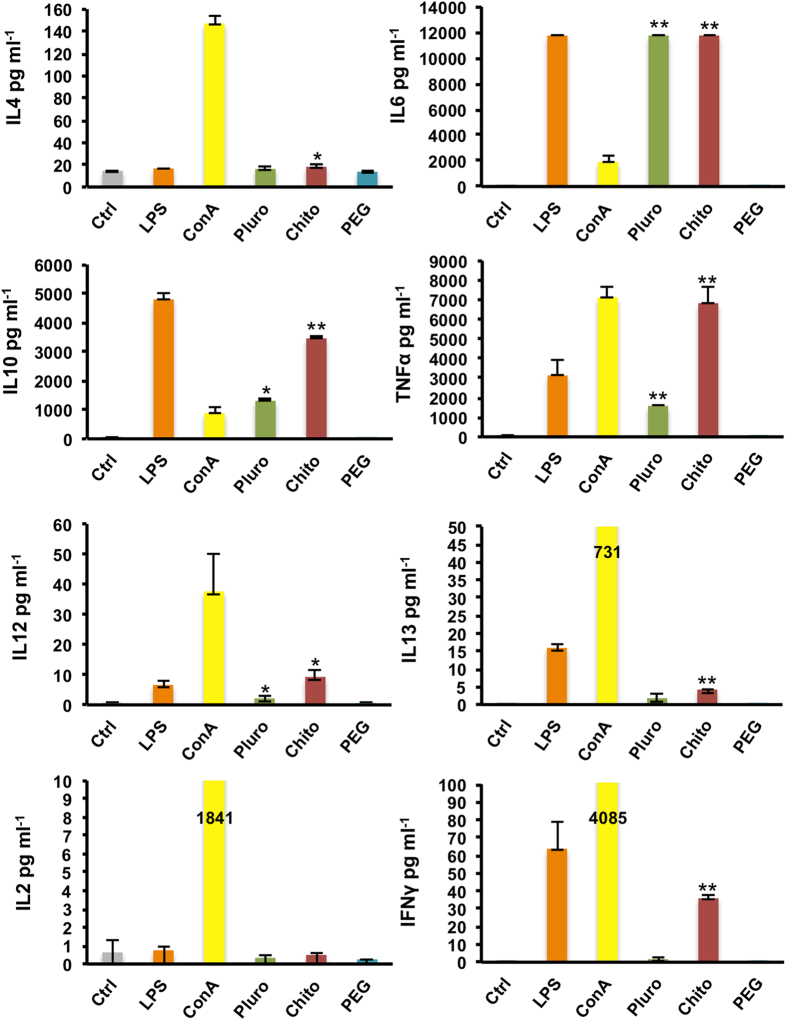
Cytokine secretion. Cytokine release was assessed by multiplex ELISA on PBMCs. Cells were incubated with nanocapsules: Pluro, Chito and PEG at dose of 1 × 10 e 11 mL^−1^ or left untreated. Concanavalin A (ConA, 10 μg/mL) and lipopolysaccharides (LPS 2 μg/mL) were used as positive controls. After 24 hour the supernatants were collected and analyzed. Supernatants were analyzed by MILLIPLEX® MAP Multiplex Assays Using Luminex xMAP® Technology. All the experiments were performed at least in triplicate (*=P value < 0.05, **=P value < 0.01).

To further investigate the Pluro, Chito and PEG NC action on immune cells, the protein level was evaluated using ELISA multiplex technology to measure the secretion of a wide variety of cytokines: IL4, IL6, IL10, TNFα, IFNγ, IL13, IL12 and IL2 (Fig. 5).

The different impact of the three functionalized NCs on immune cells was confirmed. Analytically, Fig. 5 shows that Pluro and Chito NCs have a higher effect compared to PEG NCs. Pluro NCs induced IL6, TNFα (P value < 0.01), IL12 and IL10 secretion (P value < 0.05). Moreover, Chito NCs gave a significant enhancement in IL6, IL10, TNFα, IFNγ, IL13 (P value < 0.01) IL4 and IL12 secretion (P value < 0.05). Intriguingly, PEG NCs confirmed to be perfectly inert for cells.

The cytokines found expressed such as IL6, TNFα and IL12 are normally secreted by monocytes/macrophages and their action is directed on the activation of the acute inflammation process^47^. IL12, for instance, activates NK cells and T Lymphocytes to produce IFN-γ (which we found modulated by Chito NCs) and specifically induce differentiation of T cells in T helper 1 (Th1) confirm a Th1-mediated protection enhancing also an increased cytotoxic activity^48^,^49^,^50^.

IL10, which we found secreted in both Pluro NCs (P-Value < 0.05) and Chito NCs (P-Value < 0.01), is a cytokine mainly secreted by activated macrophages that through a negative feedback directly control the immune response. IL10 plays a crucial role in the innate immunity in controlling the immune cell response, which is normally correlated with the inhibition of IL12 secretion.

Taken together these results show that Pluro and Chito NCs mediated the induction of both innate and adaptive immunity response. However, as showed in Fig. 5 the action of Pluro NCs was significantly lower than Chito NCs, taking in consideration the expression of IL10 and TNFα, 2 times and 4 times lower, respectively. This trend was also showed in the activation marker analysis on monocytes (Fig. 4B). Furthermore, Chito NCs mediated also a production of IL13 and IL4 (P-Value < 0.05). Both cytokines are strictly correlated with the activation of the lymphocyte T helper 2^51^.

## Discussion

Functionalized NCs have been developed for biomedical purposes due to many advantages: (i) improved biocompatibility and stability; (ii) extended circulation time; (iii) specific targeting function; (iv) enhanced drug encapsulation efficiency and reduced drug leakage; (v) great potential as multimodal therapeutics. All of these NC properties open new scenarios in medicine^52^.

Particularly in the context of drug delivery, our data give many positive inputs for further translational medicine research regarding the ability of the three NCs studied to pass through the immune cell membrane and to passively target the immune cells. We have previously shown that cell internalization might be useful in the context of chemotherapy by using tumor-targeted molecules^6^,^42^. Indeed, targeting capacities of these systems can be implemented by surface modification with specific molecules (*e.g.* antibodies)^14^,^53^,^54^,^55^,^56^,^57^. However, here we have demonstrated that molecules used to functionalize NCs might have diverse impacts on immune cell viability and activation, evidencing the need to know well, before any medical application, the expected action of different functionalized particles on immune cells. Pluro Chito and PEG coatings have been well investigated and characterized by many reports^20^,^58^,^59^,^60^. However, this is to our knowledge the first study comparing the action on immune cells of three highly used nanocapsule coatings for biomedical applications as it is well known in nanoscience the extreme importance of evaluating the immune response to different nanoformulations^61^. As a matter of fact, the possible interactions between nanoparticles with different coatings and the immune system, could result into an immunostimulation or an immunosuppression, which might either be applied in immunotherapy or may promote inflammatory/autoimmune disorders^62^,^63^. The main function of the immune system is to protect the host from foreign substances; however, inadvertent recognition of nanoparticles as foreign by the immune cells may result in a multilevel immune response against nanoparticles^62^. Addressing the need to carry out additional evaluation and an overall comparison of the immune impact of different nanoparticles, we aimed here at provide a broad a complete picture on the impact of differently functionalized NCs on human primary immune cells. The surface modification of systemic drug carriers by PEG is one of the preferred ways to decrease opsonization by reducing interactions with blood^64^. Recent investigations regarding PEG showed a reduced uptake and clearance by immune cells when using PEG-coated nanoparticles^20^,^58^,^65^,^66^. Jang *et al.* investigated interactions of PEG-coated grafts with embryonic immune cell lines showing that they are able to maintain stemness properties^58^. Yang *et al.* demonstrated the well-known stealth property of PEG; specifically, they showed a reduced uptake of polymeric nanoparticles by macrophages^20^. A recent report also indicate that repeated injections of PEGylated liposomes induce significant immune responses^67^. In this work the overall immune-compatibility of PEG NCs was highlighted, proving the absence of toxicity and activation stimuli. Our data may give new insights into the use of PEG coating for NCs and other nanomaterials to be used as highly safe and inert molecule^15^,^16^,^68^,^69^. A contribution was given to improve the knowledge on the absence of immune impact and toxicity of PEG in monocytes, as already reported in literature^18^,^19^ and also in primary human immune cells, total PBMCs and specifically in T cell populations. Moreover, in the context of PEG nanoparticle uptake, our PEG-PLA coating was able to bypass the well know stealth property of PEG, showing a good internalization of NCs into immune cells^20^. As it was previously proved by Stefanick JF *et al.*, this can be explained through a modulation of many parameters such as the brush conformation of the particle surface mediated by the used PEG and through a control of PEG concentration compared to PLA^39^,^40^, as it was shown by Saw P E *et al.* Notably, the length of the PEG used in this work is quite short and this may explain the higher uptake observed^33^. Moreover, Vonarbourg A *et al.* described that the stealth property of PEG NCs decreased with the increase of NC size, especially for macrophage uptake^70^. Pluro NCs showed its potential as safe coating without induced necrosis. However, data indicated an induction of apoptosis phenomena on monocytes exclusively in the 40% of cells. Our hypothesis is that Pluro NCs may have a specific action on monocytes; this is also emphasized by the membrane cell markers and cytokine analysis that showed CD69 up-modulation in monocytes and increased the release of IL6, IL10, IL12 and TNFα. The activation observed during our study might also be responsible for the apoptotic induction, a phenomenon that normally occurs during the activation process of immune cells^71^,^72^. Moreover, pluronic and chitosan were previously studied for vaccine formulations as adjuvants able to increase the immune response^73^,^74^. Our new Pluro NCs could be useful for better internalizing and delivering vaccines thanks also to their activation properties, through the specific activations of monocytes with a systemic immunization of the body enhancing the antigen presentation activity of monocytes to T Cells. The abilities of Pluro NCs on monocyte activations could be useful for drug delivery combined to immunotherapy for future new tools in cancer therapy^75^. Intriguingly, our data are not in agreement with the study of Kim HG and colleagues, as they declared that pluronic nanoparticles do not modulate immune responses mounted by macrophages. However, for their experiment that research group used a murine macrophage cell line (RAW cells) that is a good system to collect preliminary data but is not perfectly comparable with the response of human primary immune cells. Moreover, the activation that was observed in our study and particularly on cytokine secretions could be a result of various cell interactions that are possible in a PBMCs system. For this reason, further studies with Pluro NCs for its application in immunotherapy will be carried out by our research group; in fact, this may be the new frontier to fight cancer based on specific activation stimuli of immune cells against cancer cells, *i.e.* to treat melanoma, avoiding at the same time the immunosuppression of the healthy immune system induced by tumor growth^76^,^77^,^78^.

Moreover, data showed also an immune activation of cells treated with Chito NCs that is similar and even higher compared to those treated with Pluro NCs. Intriguingly, Chito NCs induced apoptosis in both populations, monocytes and T cells. The apoptotic induction of Chito NCs can be explained in the same way as for Pluro NCs; our data showed, indeed, the Chito NCs mediated induction of T cells and monocytes immune response and that action was followed by the activation of cell proliferation of both monocytes and T lymphocytes.

Cytokine analysis clarifies that after incubation with Chito and Pluro NCs, T cell activation occurred through the action of monocytes that induce the differentiation of naïve T cells in T helper cells. Thus, we observed an induced secretion of TNF and IL12 mediated by monocytes/macrophages that normally activate the T cells and NK cells to produce in the pro-inflammatory phase the same cytokine resulted increased in our experiments, such as the INF-γ which stimulates macrophages to produce TNFα. Moreover, IL12 promotes the differentiation of CD4^+^ T cells in Th1 cells that continue to produce IFN-γ as a reinforcement of macrophage immune response^48^,^49^. These cytokines organize inflammatory centers and enhance cellular immune response. With the enhancing of cytotoxic activity many intracellular pathogens can be killed through the activation of antimicrobial defenses^79^,^80^. These data confirm and extend to a human *ex vivo* system the work of Shibata Y *et al.*, where authors showed in mice that phagocytosable small-sized chitin particles activated alveolar macrophages to express cytokines such as IL-12, TNFα, and IL-18, leading to INF-γ production mainly by NK cells^81^. These results are supported also by the work of Li X *et al.*, where the systemic administration of chitosan provides significant priming effects in alveolar macrophages and NK cells in mice^59^. Interestingly, an action of Chito NCs in the secretion of IL-4 and IL-13 cytokines was found during the present study. The effector T helper 2 cells (Th2) produce normally a cytokine profile such as IL-4, IL-6, IL-10, IL-13, here were found up-regulated by Chito NCs, that together instruct B cells to proliferate and differentiate into antibody-secreting plasma cells. As such, Th2 cells play an important role providing protection against certain extracellular pathogens, such as bacteria and a variety of parasites, and are also involved in asthmatic reactions^79^,^80^. The possible activation of Th2 cells mediated by Chito NCs could be responsible for an induction of allergic response that must be well evaluated by further studies. However, the modulation of the Th response is one of the main purposes of immunotherapy and it can be useful for other therapy strategies *i.e.* in the context of vaccines^79^. As reported above for pluronic, also chitosan is currently used as an adjuvant for vaccines^59^,^82^. Nevertheless the evaluation of adjuvant properties is challenging since available data are insufficient to demonstrate immunogenicity of chitosan, its mechanism of action and to exclude impurities as the active substance^82^. Herein, we clearly reported Chito NCs mediated induction of monocytes, T cells and NK cells; specifically, we demonstrated an induction mediated by the innate immunity of Th1 able to provide a full enhancement of the immune response after treatment. All these actions occurred exclusively for Chito NCs that in our screening resulted as an excellent first step for further studies on vaccines delivery perspectives, immunotherapy and immunomodulation activities.

## Conclusion

In summary, although lipid nanocapsules harbour interesting medical applications, the potential immune impact still needs to be carefully evaluated. To our knowledge, no comprehensive studies were carried out on human immune cell populations *ex vivo* using a wide variety of functionalized NCs. In the present work, the immune cell interactions of NCs coated with pluronic, chitosan and PEG in immune cell types from healthy donors were investigated. We observed that different types of NCs lead to different impacts on immune cells and subpopulations of T cells and monocytes: totally inert (PEG NCs) or immune inductive (Pluro NCs and Chito NCs). Thanks to our data we open new windows for applications of different coated NCs in future nanomedicine-based therapies. Specifically, the Pluro NCs targeted induction of monocytes could be extremely useful for macrophage-based immunological therapies, particularly against metastases formation. In addition, the Chito-NCs-mediated induction of monocytes that activate lymphocytes Th1 response and also a Chito NCs mediate Th2 response was evidenced, providing the whole enhancement of the immune response after treatment. Based on these results, it will be useful to focus on Pluro and Chito NCs as future tools in vaccines delivery, immunotherapy and immunomodulation activities. In conclusion, our findings are of particular value towards a well-defined pre-selection of specific NC coatings based not only on their specific biomedical purposes but also in view of their immune-interaction and immuno-modulation.

## Methods

### Nanocapsule preparation

The nano-systems studied were prepared by a modified solvent displacement technique following the procedure of Calvo *et al.*^29^. Briefly, an organic phase composed of 40 mg of Epikuron 145V and 125 μL of olive oil, dissolved in 0.5 mL of ethanol and 9.5 mL of acetone, was added to 20 mL of an aqueous phase under magnetic stirring containing 50 mg of Pluronic F68 (Pluro NCs), 10 mg of chitosan (Chito NCs) or 30 mg of PEG-PLA (PEG NCs). Organic solvents (acetone and ethanol) plus a portion of the volume of water were evaporated in a rotary evaporator at 40 °C, obtaining a final volume of 15 mL. Finally, nanoparticles were extensively cleaned by dialysis through ultrapure water for 24 hours to remove the unbound surfactant molecules.

Coumarin 6-loaded lipid nanocapsules were formulated by dissolving the dye in the olive oil phase at a concentration of 0.025% (w/w). The concentration of the different nanoemulsions was estimated by calculating the volume of a single particle with the equation of the volume of a sphere: V = 4/3.π.r^3^, using the hydrodynamic mean radius obtained from DLS measurements, and then the number of particles that were formed by the total volume of olive oil were used in the synthesis.

### Size and electrophoretic mobility

A Zetasizer-nano Z (Malvern Instruments) was used to measure the hydrodynamic mean diameter and the electrophoretic mobility (μ_e_) of the nanocapsules. The μ_e_ was measured as a function of pH while maintaining a constant low-ionic-strength value (0.002 M). The size and mobility data were recorded in triplicate.

### Colloidal stability

Colloidal stability was spectrophotometrically studied monitoring the turbidity of the nanocarriers (Beckman DU 7400 spectrophotometer) working at 570 nm in simple saline media (pH 7.4). The salts used were NaCl and CaCl_2_. From the analysis of aggregation kinetics, we calculated two important parameters in colloidal-stability studies, the critical coagulation concentration (CCC), defined as the minimum salt concentration needed for the most rapid aggregation, and the critical stabilization concentration (CSC) defined as the minimum salt concentration at which the system begins to re-stabilize when salinity is progressively increased and related to the surface hydrophilicity^34^.

### Peripheral blood mononuclear cell preparation

For each experiment, human PBMCs were obtained from EDTA-venous blood samples from healthy male donors (25–50 years of age) using a standard Ficoll-Paque (GE Healtcare) separation and cultured in RPMI medium added with FBS 10% and 1% of Penicillin/Streptomycin solution. All donors provided a written informed consent. The study was reviewed and approved by the Ethics Committee of the University of Sassari. The studies were carried out in accordance with the approved guidelines.

### Uptake analysis

Uptake of coumarin 6-loaded Pluro, Chito and PEG-Nanocapsules by immune cells was studied. Nanocapsules were incubated with PBMCs for 24 hours at different concentrations (1 × 10^10^ mL^−1^, 1 × 10^11^ mL^−1^ and 1 × 10^12^ mL^−1^). Time points experiments were also conducted at 6, 12 and 24 hours with all three studied NCs, at the fixed concentration of 1 × 10^11^ mL^−1^. After incubation, cells were harvested, washed twice in cold phosphate buffered saline (PBS) and re-suspended for analysis. Cell fluorescence was measured after the cells were washed with a 0.4% (w/v) trypan blue solution in order to quench fluorescence from non-internalized NCs^17^.

The gating of cell populations was performed with specific fluorescently-labeled monoclonal antibodies: fluorescein isothiocyanate (FITC), phycoerythrin (PE), peridinin chlorophyll protein (PerCP), or allophycocyanin (APC) conjugated with anti-CD3, anti-CD4, anti-CD8, anti-CD14, anti-CD56 and anti-CD20, antibodies that were purchased from BD Biosciences (Mountain View, CA, USA). Uptake analyses were performed with flow cytometry (FACS Calibur BD Bioscience) using CellQuest software (BD Biosciences). A total of 50,000 events per sample were recorded. Data analyses and plots were performed with FlowJo software (MACS Miltenyi Biotec).

For the confocal microscopy imaging analysis, PBMCs were incubated with coumarin 6-loaded nanocapsules as described above, washed twice with PBS and re-suspended at the concentration of 2 × 10^6^ cells/0.5 mL; 30 μL of the cell solution was put onto a slide, fixed with shellac and observed by confocal laser scanning microscopy (Leica TCS SP5, Leica Microsystems). coumarin 6 was excited with an argon laser at 405 nm with 20x camera lens, the excitation filter bandwidth was: BP470/40 and the emission filter was: BP525/50. At least 20 pictures per sample for each experiments were captured.

### Hemolysis, cell viability, apoptosis and necrosis assay

Hemolysis test was conducted following previously used protocols^83^. Fresh human whole blood was taken from volunteer healthy donors stabilized with ethylenediamine tetraacetic acid (EDTA). Serum was removed from blood samples by centrifugation at 200 g for 5 minutes. Resulting RBCs were washed five times with sterile isotonic PBS and then diluted 10 X with EDTA. To determine the hemolytic activity of different NCs, Pluro, Chito and PEG NCs at the concentration of 1 × 10^11^NC mL^−1^ were added to dilute RBCs suspension (0.2 mL, 4 × 10^8^ cells/mL) in a final volume of 1mL PBS. After vortexing, the mixtures where left at room temperature for 2 hours, NCs and intact RBCs were removed by centrifugation. A microplate reader (Sunrise, Tecan) measured the absorbance (A) of the hemoglobin in the supernatant at 570 nm, with the absorbance at 620 nm as a reference.

For viability assay, PBMCs were transferred to a 24-well plate and treated for 24 hours in triplicate with Pluro, Chito, PEG nanocapsules at the concentration of 1 × 10^11^NC mL^−1^ or left untreated. Cells were treated with EtOH 70% as a positive control and then washed in cold PBS before the staining reaction. After incubation, cells were harvested, washed in cold PBS and re-suspended in 1mL of PBS at 1 × 10^6^ cells mL^−1^. Viability was assessed by the LIVE/DEAD® Fixable Dead Cell Stain Kit (Invitrogen) following the manufacturer instructions; the kit employs an amine-reactive fluorescent dye for cells with compromised membranes (late apoptotic and necrotic), the dye reacts with free amines both inside the cell and on the cell surface. In viable cells, the dye reactivity is restricted to the cell surface amines, resulting in less intense fluorescence. To detect cells undergoing apoptosis, Annexin-V FITC staining was employed (Invitrogen). Cells were analysed by flow cytometry (FACS Calibur BD-Bioscience). Data analysis was performed by flow cytometry (FACS Calibur, BD Biosciences) using CellQuest® software (BD Biosciences) and with Flowjo software (MACS Miltenyi Biotec).

### Proliferation assay

To perform the proliferation assay, the Click-iT^®^ EdU Alexa Fluor^®^ 488 Flow Cytometry Assay Kit (Life Technologies) was used. Experiments were performed according to the manufacturer’s instructions. Cells were seeded at the concentration of 1 × 10^6^ cells mL^−1^ in 96-multiwell rounded bottom plates. PBMCs were treated with Pluro, Chito, PEG NCs at the concentration of 1 × 10^11^NC mL^−1^ and Phytohemagglutinin 2% (PHA) and interleukin 2 (IL2) 30 ug/mL or left untreated. 5-ethynyl-2-deoxyuridine (EdU) were added in sterile conditions 16 hours before analysis. After 72 hours of incubation, cells were harvested, washed in PBS, fixed with 4% paraformaldehyde, washed in PBS plus 1% Bovine Serum Albumin (BSA), permeabilized with a saponin-based reagent and prepared for the Cu-assisted EdU-Azide Click-iT reaction. The reaction occurs after adding 500 μl of Click-iT reaction cocktail (PBS, CuSO_4_, fluorescent dye azide and reaction buffer additive) to each sample. Analyses were performed by Flow cytometry (FACS Calibur BD Bioscience) using CellQuest software (BD Biosciences). A total of 50, 000 events per sample were recorded.

### Activation analysis

To investigate the functional impact of Pluro, Chito, and PEG NCs on primary lymphomonocytes, considering their activation as a crucial endpoint, we previously conducted an immune cells diameter analysis. PBMCs were incubated with three NCs at the concentration of 1 × 10^11^ mL^−1^ for 24 hours. Lipopolysaccharides (2 μg/mL) were used as positive controls. After 24 hours of incubation, samples were harvested and cells were suspended in PBS. Analyses were carried out with Scepter (Millipore) using 40 μm sensor; plots and data analysis were done with Scepter Software Pro 2.1 (Millipore).

At a later stage we evaluated the expression of CD69 and CD25 activation markers by flow cytometry. Concanavalin A (4 μg/mL) or lipopolysaccharides (2 μg/mL) were used as positive controls. After 24 hours of incubation, PBMCs were stained to identify immune cell populations and analyze activation marker expression. Staining with anti-CD3, anti-CD14 anti-CD25 and anti-CD69 fluorochrome-conjugated monoclonal antibodies was performed in the dark for 20 minutes. After washing, cells were analyzed by flow cytometry (Accuri BD Biosciences). A total of 50,000 events per sample were recorded, data analysis was conducted with FlowJo software (MACS Miltenyi Biotec).

### Multiplex cytokine analysis

Cell culture supernatants from PBMCs after treatment with nanocapsules were used to quantify the secretion of cytokines using a MILLIPLEX MAP 8-plex Cytokine Kit, according to manufacturer’s protocol. Concanavalin A (ConA, 10 μg/mL) and lipopolysaccharides (LPS 2 μg/mL) were used as positive controls. The following human cytokines were measured: IL4, IL6, IL10, TNFα, IFNγ, IL13, IL12 p70 and IL2. Briefly, supernatants were centrifuged for 10 minutes to remove debris and 25 mL were added to 25 mL of assay buffer. Then, 25 mL of magnetic beads coated with specific antibodies were added to this solution and incubated for 2 hours under shaking. At the end of the incubation, the plate was washed twice in buffer and incubated for 1 hour with 25 mL of a secondary biotinylated antibody at room temperature. The plate was incubated for 30 minutes with Streptavidine Phycoerythrin, washed twice, and incubated with 150 mL of sheath fluid for 5 minutes. The plate was observed immediately on a Luminex 100/200 platform (Luminex Corporation) with xPONENT 3.1 software. Standard curves for each cytokine (in duplicate) were generated using the supplied reference cytokine concentrations. Cytokine concentrations in the samples were determined with a 5-parameter logistic curve. Final concentrations were calculated from the mean fluorescence intensity and expressed in pg/mL. All incubation steps were performed at room temperature and in the dark.

## Additional Information

**How to cite this article**: Farace, C. *et al.* Immune cell impact of three differently coated lipid nanocapsules: pluronic, chitosan and polyethylene glycol. *Sci. Rep.* **6**, 18423; doi: 10.1038/srep18423 (2016).

## Supplementary Material

Supplementary Information
