# Supplementary material for: Immune cell impact of three differently coated lipid nanocapsules: pluronic, chitosan and polyethylene glycol
Source: Sci Rep. 2016 Jan 5;6:18423. doi: 10.1038/srep18423 (PMC4700454; doi:10.1038/srep18423)
Supplement: Supplementary Information [file srep18423-s1.pdf]

Immune cell impact of three differently coated lipid nanocapsules: pluronic, chitosan and polyethylene glycol

Cristiano Farace[1,‡], Paola Sánchez-Moreno[2,‡], Marco Orecchioni[3], Roberto Manetti[3], Francesco Sgarrella[3], Yolande Asara[1], José M. Peula-García[5,6], Juan A. Marchal[7,8,9], Roberto Madeddu[1,10] and Lucia G. Delogu[2]*.*

*=**Corresponding Authors:** E-mail: lgdelogu@uniss.it, rmadeddu@uniss.it

Supplementary Information

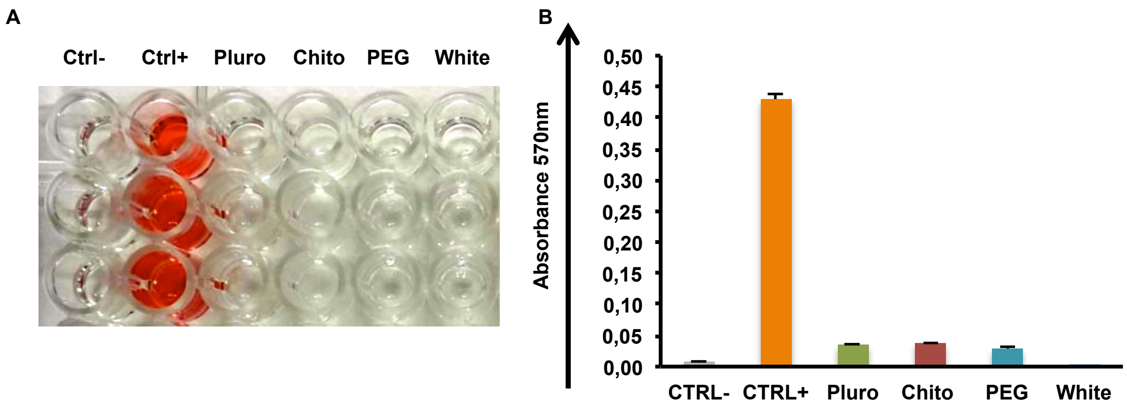

S. Figure 1. Hemolysis. Pluro, Chito, PEG-NCs ($1x10^{11}$ mL^{-1} concentration) were incubated with red blood cells (RBCs) at 25°C for 2 h. (A) Pictures of human RBCs treated with Pluro, Chito and PEG NCs. The red color of the solution is due to the release of hemoglobin from the damaged RBCs. PBS mixed with RBCs (Ctrl-) and ultrapure water mixed with RBCs (Ctrl+) served as negative and positive control respectively, PBS alone was used as additional control (white). (B) Sample Absorbance at the wavelength of 570nm-620nm. Experiments were performed in triplicate.

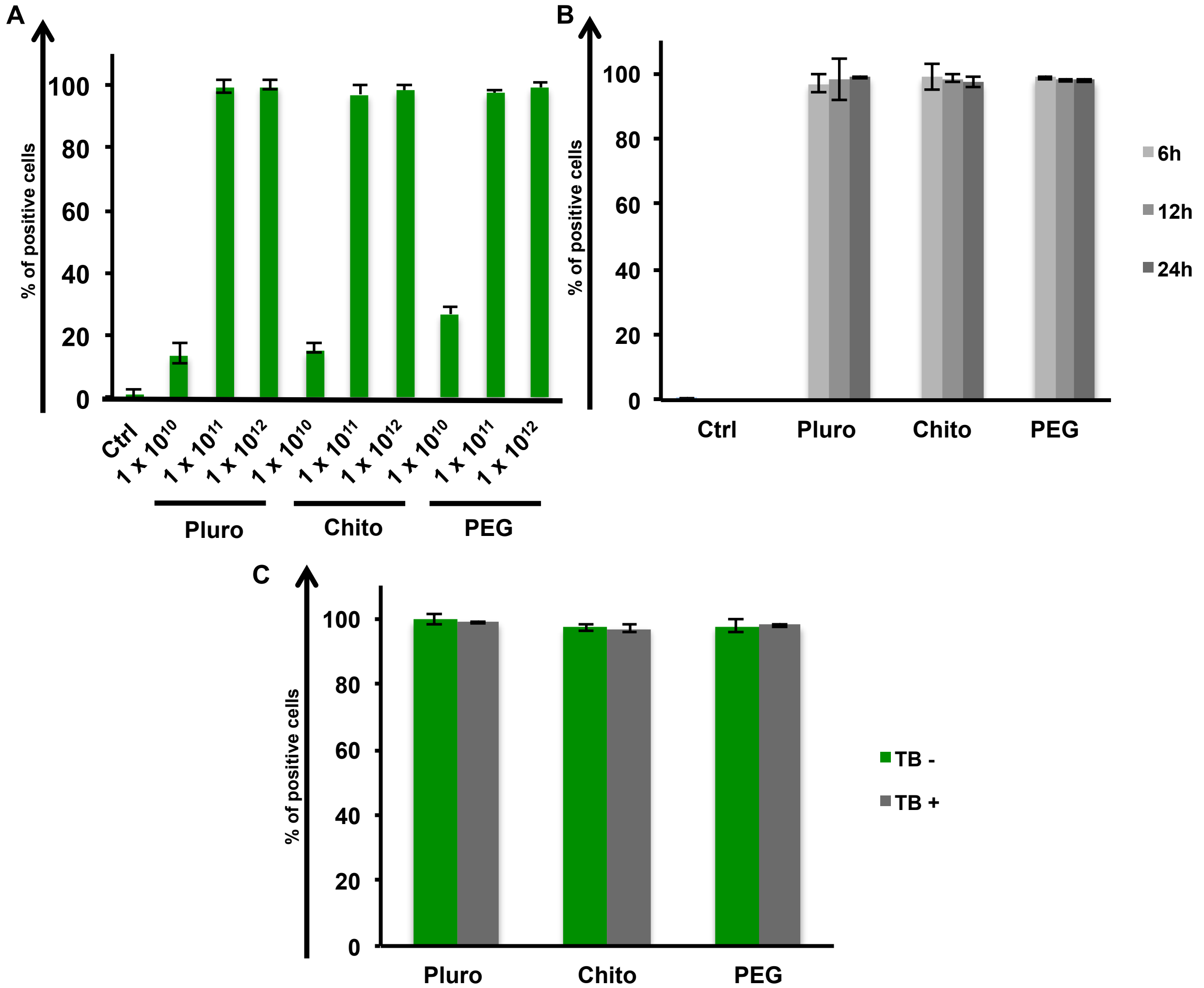

S. Figure 2. Uptake analysis. A) PBMCs were either left untreated or incubated for 24 h with $1x10^{10}$ mL^{-1} , $1X10^{11}$ mL^{-1}, $1X10^{12}$ mL^{-1} of three different functionalized nanocapsules (Pluro, Chito, Peg) with Courmarin 6. **B)** Uptake analysis at different time points 6h, 12h and 24h of three NCs at the concentration of $1X10^{11}$ mL^{-1} Trypan Blue (TB) was used as a quencher for non internalized NCs. As a control we used the untreated cells. **C**) Uptake comparison between cells washed and unwashed with TB after 24h of incubation with the three NCs at the concentration of $1X10^{11}$ mL^{-1}. Experiments performed in triplicate were analyzed by Flow Cytometry and FlowJo software.

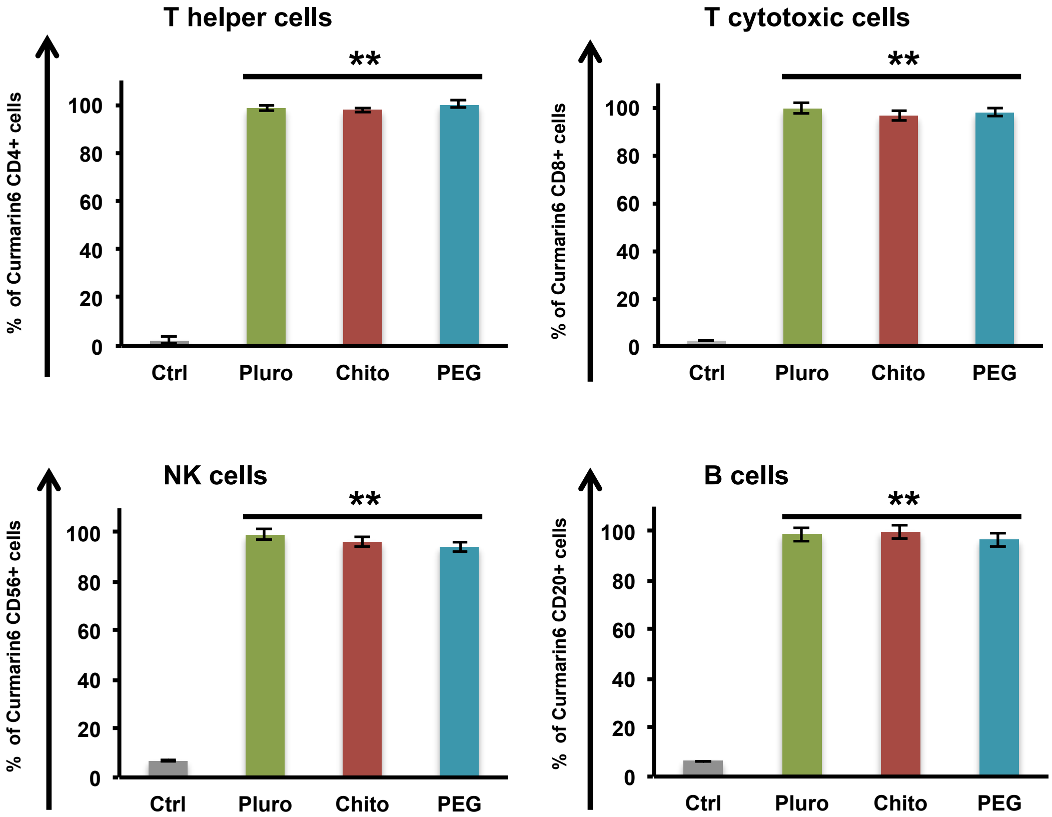

S. Figure 3. Uptake analysis on PBMC subpopulations. NCs uptake assessment on lymphocytes T helper (CD4+), lymphocytes T cytotoxic (CD8+), Natural killer (CD56+) and B cells (CD20+). Cells were incubated for 24 hour with Pluro, Chito and PEG NCs at the concentration of at $1x10^{11}$ mL^{-1} or left untreated, samples were analysed by flow cytometry. Experiments were performed at least in triplicate. **= P value<0,01.
